# Estimating Error in Using Residential Outdoor PM_2.5_ Concentrations as Proxies for Personal Exposures: A Meta-analysis

**DOI:** 10.1289/ehp.0901158

**Published:** 2010-01-14

**Authors:** Christy L. Avery, Katherine T. Mills, Ronald Williams, Kathleen A. McGraw, Charles Poole, Richard L. Smith, Eric A. Whitsel

**Affiliations:** 1 Department of Epidemiology, University of North Carolina–Chapel Hill, Chapel Hill, North Carolina, USA; 2 U.S. Environmental Protection Agency, National Exposure Research Laboratory, Research Triangle Park, North Carolina, USA; 3 Health Sciences Library; 4 Department of Statistics and Operations Research and; 5 Department of Medicine, University of North Carolina–Chapel Hill, Chapel Hill, North Carolina, USA

**Keywords:** air pollution, measurement error, meta-analysis, PM_2.5_

## Abstract

**Background:**

Studies examining the health effects of particulate matter ≤ 2.5 μm in aerodynamic diameter (PM_2.5_) commonly use ambient PM_2.5_ concentrations measured at distal monitoring sites as proxies for personal exposure and assume spatial homogeneity of ambient PM_2.5_. An alternative proxy—the residential outdoor PM_2.5_ concentration measured adjacent to participant homes—has few advantages under this assumption.

**Objectives:**

We systematically reviewed the correlation between residential outdoor PM_2.5_ and personal PM_2.5_ (*r̄_j_*) as a means of comparing the magnitude and sources of measurement error associated with their use as exposure surrogates.

**Methods:**

We searched seven electronic reference databases for studies of the within-participant residential outdoor-personal PM_2.5_ correlation.

**Results:**

The search identified 567 candidate studies, nine of which were abstracted in duplicate, that were published between 1996 and 2008. They represented 329 nonsmoking participants 6–93 years of age in eight U.S. cities, among whom *r̄_j_* was estimated (median, 0.53; range, 0.25–0.79) based on a median of seven residential outdoor-personal PM_2.5_ pairs per participant. We found modest evidence of publication bias (symmetric funnel plot; *p*_Begg_ = 0.4; *p*_Egger_ = 0.2); however, we identified evidence of heterogeneity (Cochran’s *Q*-test *p* = 0.05). Of the 20 characteristics examined, earlier study midpoints, eastern longitudes, older mean age, higher outdoor temperatures, and lower personal-residential outdoor PM_2.5_ differences were associated with increased within-participant residential outdoor-personal PM_2.5_ correlations.

**Conclusions:**

These findings were similar to those from a contemporaneous meta-analysis that examined ambient-personal PM_2.5_ correlations (*r̄_j_* = median, 0.54; range, 0.09–0.83). Collectively, the meta-analyses suggest that residential outdoor-personal and ambient-personal PM_2.5_ correlations merit greater consideration when evaluating the potential for bias in studies of PM_2.5_-mediated health effects.

Numerous epidemiologic and toxicologic studies have linked particulate matter (PM) air pollution with adverse health outcomes, including mortality (Burnett et al. 2000; Dominici et al. 2003; Katsouyanni et al. 2003), hospital admissions (Burnett et al. 1995; Linn et al. 2000; Oftedal et al. 2003), and subclinical disease (Diez Roux et al. 2008; Liao et al. 2009; Whitsel et al. 2009). A common feature of such studies is their reliance on ambient PM concentrations measured at distal monitoring sites as proxies for personal exposure to PM of ambient origin. The reliance is consistent with regulatory policies developed under the Clean Air Act (1970) which have been informed by studies of the correlation between personal exposures to PM originating outdoors and residential outdoor PM concentrations (Wallace 2000). However, ambient PM may not adequately represent total PM exposure, because human activity pattern surveys suggest that, on average, individuals spend > 85% of their time inside (Klepeis et al. 2001), where they are exposed to numerous sources of indoor PM, the physicochemical properties and toxicities of which often differ from those of ambient PM (Monn and Becker 1999; Wainman et al. 2000).

Available exposure studies, although small in number, have suggested that several factors may influence the relationship between ambient and total PM exposure, including home ventilation, indoor PM sources, and time–activity patterns (Rodes et al. 2001; Sarnat et al. 2006; Williams et al. 2003b). Because these factors are not well quantified (Janssen et al. 1998), we previously reviewed the literature that examined the within-participant ambient-personal PM_2.5_ correlation to determine the magnitude and sources of measurement error inherent in using ambient PM_2.5_ as a surrogate for personal exposure (Avery et al. 2010). We found that characteristics of participants, studies, and the environments in which they were conducted affect the accuracy of ambient PM_2.5_ as a proxy for personal exposure and that the potential for exposure misclassification may be substantial.

Although the residential outdoor PM_2.5_ concentration measured adjacent to participant homes may be equally prone to misclassification under the assumption of spatial homogeneity, use of this measure as an alternative proxy for personal exposure may have some advantages if this assumption is not uniformly applicable. Studies of spatial variability in ambient PM_2.5_ concentrations among 27 U.S. urban areas (Pinto et al. 2004) suggest that this may be the case. The fact that PM_2.5_ varies at the microenvironmental level as a function of, for example, topography, proximity to PM_2.5_ point sources, adjacency to major traffic arterials, and prevailing winds [U.S. Environmental Protection Agency (EPA) 2009; Zhu et al. 2002] also is consistent with this suggestion. Nonetheless, how spatial variability and outdoor microenvironments affect the use of ambient PM_2.5_ concentrations as a proxy for personal PM_2.5_ exposure remains unclear. Thus, we performed a meta-analysis using the literature that examined the within-participant residential outdoor-personal PM_2.5_ correlation and contrasted these findings with those from the review of the within-participant ambient-personal PM_2.5_ correlation (Avery et al. 2010). Findings from the two meta-analyses will facilitate the quantification of bias that resulted from the use of surrogates for personal PM_2.5_ exposure in studies that relied on outdoor PM_2.5_ measurements.

## Methods

### Systematic review strategy

We devised a search strategy to identify studies of the within-participant residential outdoor-personal PM_2.5_ correlation. No limitations on document type, language, or publication date were used. On 12 November 2007, we conducted searches in PubMed (http://www.ncbi.nlm.nih.gov/pubmed; 1950 to 12 November 2007), Web of Science (http://thomsonreuters.com/products_services/science/science_products/a-z/web_of_science; 1955 to 12 November 2007), BIOSIS Previews (http://www.thomsonscientific.com/cgi-bin/jrnlst/jloptions.cgi?PC=BP; 1969 to 12 November 2007), CSA Environmental Sciences and Pollution Management (http://www.csa.com/factsheets/envclust-set-c.php; 1967 to 12 November 2007), TOXLINE (http://toxnet.nlm.nih.gov/; 1965 to 12 November 2007), and Proquest Dissertations and Theses (http://www.proquest.com/en-US/catalogs/databases/detail/pqdt.shtml; 1861 to 12 November 2007). We searched EMBASE (http://www.embase.com/; 1974 to 12 November 2007), on 14 December 2007.

The following strategy was used to search PubMed: (PM 2.5 OR PM2.5 OR PM25 OR PM 25 OR fine particle) AND (ambient OR outdoor OR outdoors OR outside OR exterior OR external OR background OR fixed site*) AND (individual OR personal) AND (correlat* OR associat* OR relat* OR compar* OR pearson OR spearman). The same four sets of key words were adapted for input into Web of Science, BIOSIS Previews, CSA Environmental Sciences and Pollution Management, TOXLINE, and EMBASE. The Dissertations and Theses search required only the first three sets of key words to create a result set small enough for review.

We downloaded citations to an electronic reference manager (EndNote X1; Thomson Reuters, New York, NY), de-duplicated, and supplemented with secondary references cited in articles identified in the primary search. The citations were independently reviewed with respect to three inclusion criteria: measurement of residential outdoor PM_2.5_, measurement of personal PM_2.5_, and estimation of the within-participant residential outdoor-personal PM_2.5_ correlation. Study, participant, and environment characteristics were extracted from all articles meeting the inclusion criteria. The study characteristics were journal of publication, publication date, setting, study dates, sample size, duration of study, timing (consecutive, nonconsecutive), lower limit of PM_2.5_ detection, number (minimum, mean) of paired PM_2.5_ measures, and correlation metric (Pearson, Spearman). Participant characteristics included age (mean, minimum, maximum), percent female, and the presence of comorbidities (pulmonary, cardiovascular, multiple, neither). Environmental characteristics included the mean, median, and standard deviation of PM_2.5_ concentrations (residential outdoor, personal), the within-participant residential outdoor-personal PM_2.5_ correlation coefficients and corresponding number of paired measurements, season, distance to monitor, monitor type, air exchange rate, percentage of time using air conditioning, and percentage of time with windows open. Discrepant exclusions and extractions were adjudicated by consensus. Supplemental data were requested from authors by electronic mail as needed. City-specific longitudes and latitudes were obtained from the GEOnet Names Server (National Geospatial-Intelligence Agency 2009). Meteorologic data were obtained from the National Climatic Data Center (2009).

### Statistical analysis

Summary correlation and variance estimates for the *j*th study were estimated from the personal ambient PM_2.5_ correlations measured for each of the *i*th participants. Each within-participant correlation coefficient (*r_i_*) was converted to its variance-stabilizing Fisher’s *z*-transform: *Z_r_i__* = (1 ÷ 2)log*_e_*[(1 + *r_i_*) ÷ (1 − *r_i_*)] (Fisher 1925). Estimates of the within-participant variance [*v_i_* = 1 ÷ (*n_i_* − 3)] and between-participant variance (τ*_j_*^2^ = [*Q_j_* − (*k_j_* − 1)] ÷ *c*) for the *j*th study were estimated from the number of paired personal-residential outdoor PM_2.5_ measurements for each participant (*n_i_*), the number of participants per study (*k_j_*), the weighted sum of squared errors [*Q_j_* = ∑*_i_*_=1_*^k^* (*n_i_* − 3)(*Z_r_i__* − *Z_r_i__*)^2^], and a constant (*c*) = ∑*^k^_i_*_=1_(*n_i_* − 3) − [∑*^k^_i_*_=1_(*n_i_* − 3)^2^ ÷ ∑*_i_*_=1_*^k^* (*n_i_* − 3)]). The transformed effect size for the *j*th study is given by *Z̄_j_* = ∑*_i_*_=1_*^k^w_i_Z_r_i__* ÷ ∑*_i_*_=1_*^k^w_i_* with participant-specific weights [*w_i_* = ([1 ÷ (*n_i_* − 3)] + τ*_j_*^2^)^−1^], study-specific standard errors [*S_j_* = (1 ÷ ∑*_i_*_=1_*^k^w_i_*)^1/2^], and study-specific weights [*W_j_* = (1 ÷ *s_j_*)^2^]. Negative τ^2^ estimates were set to 0 (Field 2001).

We assessed publication bias, which is present when study results influence the chance or timing of publication (Begg and Berlin 1989), using a “funnel plot” of *W_j_* versus *Z̄_j_*. In the absence of publication bias, plots usually resemble a symmetrical funnel, with the more precise estimates forming the spout and the less precise estimates forming the cone. We also evaluated the adjusted rank correlation (Begg and Mazumdar 1994) and regression asymmetry tests (Egger et al. 1997) as well as a nonparametric “trim-and-fill” method that imputes hypothetically missing results due to publication bias (Duval and Tweedie 2000). Low *p*-values associated with the former tests (*p*_Begg_, *p*_Egger_) give evidence of asymmetry.

Interstudy heterogeneity was evaluated using a plot of *Z̄_j_* ÷ *S_j_* versus 1 ÷ *S_j_* (Galbraith 1988) and with Cochran’s *Q*-test (Cochran 1954). The plot and test are related in that the position of the *j*th study along the vertical axis illustrates its contribution to *Q*-test statistic. In the absence of appreciable evidence of heterogeneity, all studies fall within the 95% confidence interval (CI) and *p*_Cochran_ > 0.1.

We first assessed variation in the strength and precision of *Z̄_j_* across levels of the study, environment, and participant characteristics with a summary random-effects estimate of *Z̄* within each study, environment, and participant category (Berkey et al. 1995). We also constructed a series of univariable random-effects meta-regression models to relate each study, environment, and participant characteristic to differences in -*Z̄_j_*. Lastly, a multivariable random-effects meta-regression model and a backward elimination strategy were used to evaluate 8 study, participant, and environment characteristics routinely available in epidemiologic studies of PM_2.5_ health effects: latitude, longitude, mean age, percent female, relative humidity, sea level pressure, mean temperature, and mean residential outdoor PM_2.5_ (measured in this setting or spatially interpolated in other studies). Interval-scale characteristics were analyzed before and after dichotomization at their medians unless noted otherwise. We used STATA (version 9; StataCorp LP, College Station, TX) to perform all the analyses. To facilitate interpretation, summary estimates (i.e., *Z̄*) were back-transformed to their original metric *r̄* after data analysis.

## Results

The systematic review identified 567 candidate studies for screening. Of these studies, nine (2%) met the criteria for critical appraisal and were abstracted (Brown et al. 2008; Liu et al. 2003; Reid 2003; Rodes et al. 2001; Rojas-Bracho et al. 2000; Suh et al. 2003; Wallace 1996; Williams et al. 2000a, 2000b, 2003a). Abstracted studies were published between 1996 and 2008 (Table 1), were set in eight cities in six U.S. states, and were conducted between 1989 and 2001. The median study duration was 1.9 months (range, 0.2–15.2 months), a period in which 70% of the studies collected PM_2.5_ data over consecutive days. During data collection, the investigators recorded a median of seven (range, 5–20) pairs of residential outdoor and personal PM_2.5_ concentrations per participant, on which the within-participant Pearson (63%) and Spearman (37%) correlation coefficients were based (Table 1).

**Figure 1:**
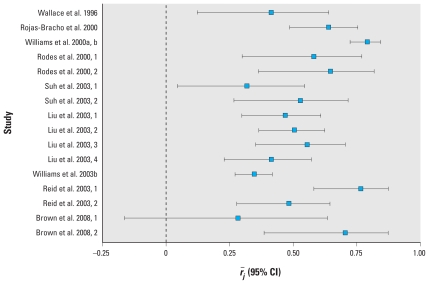
Forest plot for 16 estimates of *r̄_j_* (95% CIs) from nine studies of the within-participant residential outdoor-personal PM_2.5_ correlation.

The studies represented 329 nonsmoking participants 6–93 (median, 70) years old, 55% of whom were female and 25% of whom did not report chronic pulmonary or cardiovascular disease (Table 2). On average, residential outdoor PM_2.5_ concentrations (range, 8.6–42.6 μg/m^3^) were lower than personal PM_2.5_ concentrations (range, 9.3–70.0 μg/m^3^), with a median residential outdoor-personal PM_2.5_ difference of −1.55 μg/m^3^ (range, −27.4 to 9.0 μg/m^3^; Table 3). The estimated *r̄_j_* (median, 0.53; range, 0.25–0.79) and its standard deviation varied widely (Figure 1), the latter reflecting variability in sample weights (median, 53.6; range, 9.4–548.1). Temperature (range, 2.0–24.0°C) and relative humidity (range, 27.3–78.9%) were also variable.

**Figure 2:**
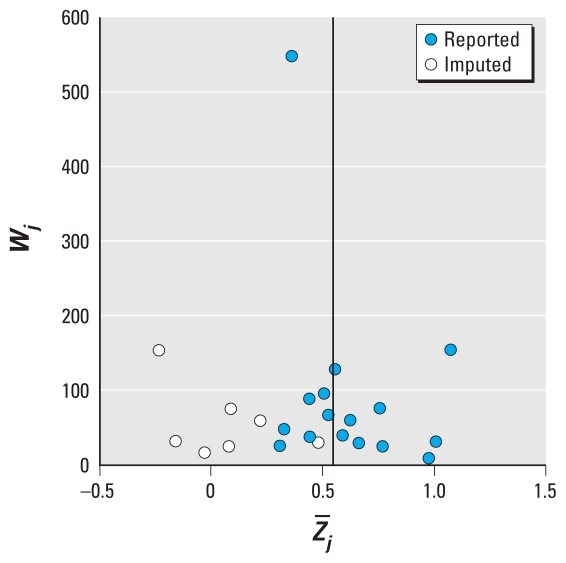
Funnel plot for 16 estimates of the within-participant residential outdoor-personal PM_2.5_ correlation.

**Figure 3:**
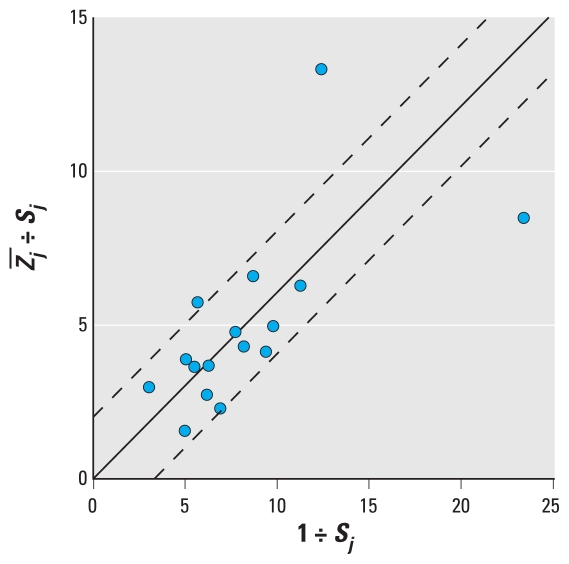
Galbraith plot with 95% CIs for 16 estimates of the within-participant residential outdoor-personal PM_2.5_ correlation.

Figure 2, a funnel plot of *Z̄_j_*, shows little evidence of asymmetry. This was consistent with *p*_Begg_ = 0.4, *p*_Egger_ = 0.2, although the “trim-and-fill” analysis imputed seven hypothetically missing studies. Figure 3, a Galbraith plot in which three observations fell outside the 95% CIs, provides evidence of heterogeneity. This evidence was consistent with *p*_Cochran_ = 0.05.

**Figure 4:**
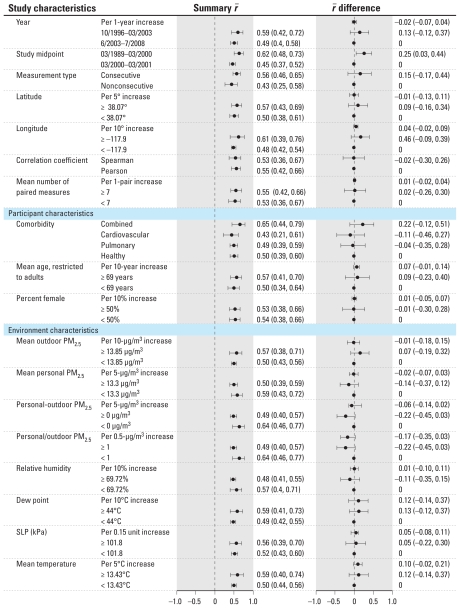
Unadjusted summary correlations (95% CIs) and differences (95% CIs) by study, participant, and environment characteristics for nine studies examining the within-participant residential outdoor-personal PM_2.5_ correlation. Summary correlations represent stratum-specific estimates of *r̄*. Increases in *r̄* per unit change of study, participant, and environment characteristics are provided by *r̄* difference estimates. SLP, sea level pressure.

**Figure 5:**
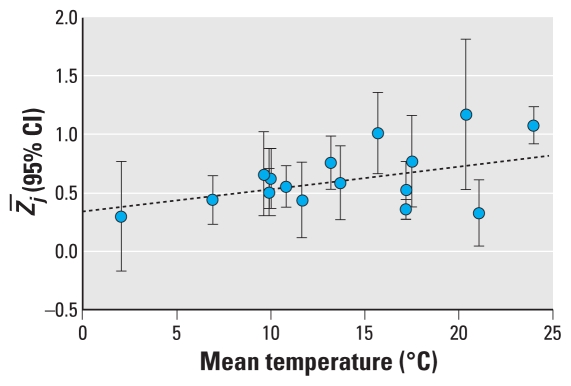
Plot for 16 estimates of the within-participant residential outdoor-personal PM_2.5_ correlation (95% CI) versus mean outdoor temperature, including the univariate random-effects meta-regression line.

Several study, participant, and environmental characteristics were suggestively associated with moderate increases in the within-participant residential outdoor-personal PM_2.5_ correlation coefficient in univariate meta-regression models (Figure 4), including earlier study midpoints, eastern longitudes, older mean age, lower personal-residential outdoor PM_2.5_ differences (and ratios), and higher mean temperatures (Figure 5). For example, every 5°C increase in mean temperature was associated with a 0.10 95% CI, (−0.02, 0.21) unit difference in *r̄*. The direct association between mean temperature and *r̄_j_* also was apparent when evaluating mean temperature dichotomized at the median: In studies with a mean temperature ≥ 13.43°C, *r̄* was 0.59 (range, 0.40–0.74), and in those with a mean temperature < 13.43°C, *r̄* was 0.50 (range, 0.44–0.56).

When evaluating multivariable meta-regression models, only higher mean ages and eastern longitudes were associated with an increased within-participant residential outdoor-personal PM_2.5_ correlation coefficient (*p* < 0.05).

## Discussion

Epidemiologic studies of the health effects of PM_2.5_ typically estimate PM_2.5_ exposures using daily mean concentrations either obtained from a single ambient PM_2.5_ monitoring site or averaged across several sites (U.S. EPA 1996). Although rapid dispersion and secondary formation of atmospheric PM_2.5_ via chemical reactions of such gases as sulfur dioxide, nitrogen oxides, and ammonia ensure some geographic uniformity of the monitored concentrations, primary sources of anthropogenic PM_2.5_, including traffic, construction, and industry (Samet and Krewski 2007), can increase the spatial variability of PM_2.5_. Additional factors that influence the relationship between ambient PM_2.5_ concentrations and PM_2.5_ exposures include home ventilation, indoor activities associated with generation or resuspension of PM_2.5_ like cooking or cleaning, and time–activity patterns (Liu et al. 2003; Williams et al. 2000b). Thus, estimates of PM_2.5_ exposure based on ambient PM_2.5_ concentrations are associated with an acknowledged degree of uncertainty (Janssen et al. 1998).

To further characterize this uncertainty, in the present study we extended a prior meta-analysis of the within-participant ambient-personal PM_2.5_ correlation (Avery et al. 2010) by examining the within-participant residential outdoor-personal PM_2.5_ correlation using analogous meta-analytic methods. In both cases, the examination generated little evidence for publication bias of Fisher’s *z*-transformed *r̄_j_* but strong evidence of heterogeneity. Several study, participant, and environment characteristics were associated with an increased *r̄_j_*, including earlier study midpoints, eastern longitudes, lower personal-residential outdoor PM_2.5_ differences (and ratios), higher mean ages, and higher mean temperatures. Moreover, the direct association between eastern longitudes and increased *r̄_j_* was consistent with the prior meta-analysis of the within-participant ambient-personal PM_2.5_ correlation.

The direct association between eastern longitudes and increased *r̄_j_* may reflect several regional factors, including higher urban PM_2.5_ concentrations (Rom and Markowitz 2006) or a greater influence of secondary PM_2.5_ sources in eastern locales (Pinto et al. 2004). The inverse associations between the residential outdoor-personal PM_2.5_ difference (or ratio) and mean temperature with *r̄_j_* may also suggest lower microenvironmental variation in PM_2.5_ or an increased contribution of residential outdoor to personal PM_2.5_ exposure, through either time–activity patterns or increased air exchange. We were unable to fully evaluate the influence of these factors given the limited number of published studies and their inconsistent reporting of other geographic, household, and personal factors potentially responsible for the above associations. However, higher mean ages and eastern longitudes were associated with increased *r̄_j_* in the multivariable prediction model that included study, participant, and environment characteristics routinely available in epidemiologic studies of PM_2.5_ health effects.

Although the meta-analyses of the ambient-personal and residential outdoor-personal PM_2.5_ correlations summarized a wide range of published correlation coefficients, both of them estimated a median *r̄_j_* of 0.5, which suggests that attempting to account for spatial variability and outdoor microenvironments does not appreciably affect the use of outdoor PM_2.5_ concentrations as proxies for personal PM_2.5_ exposure in the settings examined by the source studies. Nonetheless, these simple measures of central tendency have potentially important implications for studies using PM_2.5_ concentrations measured at distal or proximal monitoring sites. For example, an *r̄* of 0.5 implies that, on average, only *r̄*^2^ or one-fourth of the variation in personal PM_2.5_ is explained by ambient or residential outdoor PM_2.5_ concentrations. Under a simple measurement error model, it also implies that the variances of ambient or residential outdoor PM_2.5_ concentrations are 1/*r̄*^2^, or four times as large as the variance of the true, but often unmeasured, personal PM_2.5_ exposure. Moreover, *r̄* values of 0.5 in diseased and nondiseased subpopulations (i.e., nondifferential exposure measurement error) imply that *a*) sample sizes needed to detect between-group differences in mean ambient or residential outdoor PM_2.5_ concentrations are 1/*r*^2^, or 4-fold as large as those needed to detect the same differences in personal PM_2.5_ exposures, and *b*) effect estimates expressed as microgram per cubic meter increases in ambient or residential outdoor PM_2.5_ concentrations are equal to those associated with the same microgram per cubic meter increases in personal PM_2.5_ exposure, albeit attenuated toward the null by the power *r*^2^ or 0.25. The latter form of attenuation is capable of obscuring weak to modest health effects of PM_2.5_ (White et al. 2003), yet it cannot be adequately controlled by methods commonly used to account for confounding (Greenland and Robins 1985).

Given the above considerations, it is tempting to assume that all health effect estimates based on ambient or residential outdoor PM_2.5_ concentrations would be considerably larger if they were instead based on personal PM_2.5_ exposures, but to do so would yield more biased estimates if the original PM_2.5_–disease associations were spurious due to chance or confounding (Armstrong 1998). This justifies the application of the present findings to the PM_2.5_–disease associations that are the most precise and least biased according to criteria used to judge epidemiologic evidence (Hill 1965; Poole 2001; U.S. EPA 2009). Furthermore, factors associated with *r̄*, such as mean age and eastern longitudes, may differ among participants and the studies in which they are enrolled. It is therefore difficult to predict the degree to which PM_2.5_ health effects estimates may be biased by exposure measurement error. Nonetheless, the above examples clearly illustrate that the impact of *r̄* on the interpretation of findings from studies of PM_2.5_ health effects may be substantial.

Although in the present study we attempted to quantify the error associated with using residential outdoor and ambient PM_2.5_ concentrations as proxies for total personal exposure, the approach adopted here has several limitations. First, residential outdoor and ambient PM_2.5_ concentrations are likely to be poor proxies for exposure to nonambient PM because PM originating indoors has different compositions and biological properties (Long et al. 2001). Although the relative toxicity of outdoor and indoor PM remains under investigation, a panel study of 16 chronic obstructive pulmonary disease patients in Vancouver, British Columbia, reported that only the PM originating outdoors was associated with adverse cardiopulmonary effects (Ebelt et al. 2005). Moreover, in the present study we did not evaluate the correlation between concentrations of PM originating almost exclusively outdoors (e.g., sulfate or elemental carbon) and personal PM_2.5_ exposure, despite reports that their associations with ambient PM_2.5_ are particularly strong (Ebelt et al. 2000; Sarnat et al. 2006). Further work examining the relative contributions of PM_2.5_ constituents to PM-mediated health effects is clearly needed.

In summary, the results presented here and in the previous meta-analysis of the within-participant ambient-personal PM_2.5_ correlation (Avery et al. 2010) suggest that greater scrutiny of the effects of exposure measurement error is warranted. Further inquiry should involve quantifying the impact of using ambient or residential outdoor PM_2.5_ concentrations as proxies for personal PM_2.5_ exposure, as well as the development of methodologies to apply such findings. A comprehensive understanding of the degree to which these proxies influence PM_2.5_–disease associations is especially important in air pollution epidemiology because the health effects of PM_2.5_ exposure may be subtle. Such subclinical effects are particularly difficult to detect in the presence of measurement error because sensitivity of detection varies inversely with the degree of misclassification (Rom and Markowitz 2006).

## Figures and Tables

**Table 1:** Characteristics of nine U.S. studies examining the within-participant residential outdoor-personal PM_2.5_ correlation.

			Study dates (month/day/year)					
	Setting		Start	End	Duration (months)	PM_2.5_ measures		
Study/substudy	City	State				Timing	Pairs	*r*
Wallace 1996	Azusa	CA	03/06/1989	03/13/1989	0.2	N	7	P
Rojas-Bracho et al. 2000	Boston	MA	02/05/1996	02/02/1997	11.7	C	13	P
Williams et al. 2000a, 2000b	Towson	MD	07/26/1998	08/23/1998	0.9	C	16	P
Rodes et al. 2001								
1	Fresno	CA	02/01/1999	02/28/1999	0.9	C	8	P
2	Fresno	CA	04/19/1999	05/16/1999	0.9	N	7	P
Suh et al. 2003								
1	Los Angeles	CA	06/12/2000	07/24/2000	1.4	C	6	S
2	Los Angeles	CA	02/11/2000	03/22/2000	1.3	C	6	S
Liu et al. 2003								
1	Seattle	WA	10/26/1999	08/10/2000	9.3	C	7	P
2	Seattle	WA	10/26/1999	10/26/2000	11.8	C	7	P
3	Seattle	WA	02/07/2000	05/24/2001	15.2	C	7	P
4	Seattle	WA	11/27/2000	02/24/2001	2.9	C	7	P
Reid 2003								
1	Atlanta	GA	09/21/1999	11/23/1999	2.0	C	6	S
2	Atlanta	GA	04/01/2000	05/13/2000	1.4	C	6	S
Williams et al. 2003a	Raleigh	NC	06/09/2000	05/21/2001	11.2	N	20	P
Brown et al. 2008								
1	Boston	MA	11/15/1999	01/29/2000	2.4	C	6	S
2	Boston	MA	06/06/2000	07/25/2000	1.6	C	5	S
All nine studies totaled (1996–2008), 16 substudies	8	6	1989 – 2001	1.9	70% C	7	63% P

Abbreviations: C, consecutive; N, nonconsecutive; P, Pearson product-moment correlation coefficient; *r*, within-participant residential outdoor-personal PM_2.5_ correlation estimation method; S, Spearman’s rank correlation coefficient. Summary statistics are reported as counts, range, proportion, or median. “Pairs” indicates average number of outdoor-personal paired measurements for estimation of within-participant correlations. Williams et al. 2000a and 2000b refer to the same study.

**Table 2:** Characteristics of participants in nine studies that examined the within-participant residential outdoor-personal PM_2.5_ correlation.

			Participant Age				
Study	Substudy	*n*	Mean	Minimum	Maximum	Percent female	Comorbidity^a^
Wallace 1996		10	34.1	11	52	30	N
Rojas-Bracho et al. 2000		17	—b	—b	—b	—b	P
Williams et al. 2000a, 2000b		19	81	72	93	81	N, C, P
Rodes et al. 2001	1	5	85	55	—b	68	N
	2	14	85	55	—b	68	N
Suh et al. 2003	1	14	68.1	55	84	87	P
	2	13	70	60	84	93	P
Liu et al. 2003	1	30	76.3	66	88	61	N
	2	48	77.3	65	89	55	P
	3	33	76.6	57	86	35	C
	4	22	9	6	13	24	P
Reid 2003	1	23	64	33	88	33	C, P
	2	22	63	33	84	50	C, P
Williams et al. 2003a		36	70	55	85	74	C
Brown et al. 2008	1	12	—c	40	—c	20	C, P
	2	11	—c	40	—c	27	C, P
All nine studies totaled 1996–2008	16	329	70	6	93	55%	25% N

Abbreviations: N, no disease; P, chronic pulmonary disease; C, chronic cardiovascular disease.

a Summary statistics reported as counts, range, proportion, or median;

b Requested but not provided as of 18 November 2009.

c Not collected. Williams et al. 2000a and 2000b refer to the same study.

**Table 3:** Environmental characteristics for nine studies that examined the within-participant correlation between residential outdoor and personal PM_2.5_.

		Residential outdoor PM_2.5_ (μg/m^3^)	Personal PM_2.5_ (μg/m^3^)	*r*		Meteorologic data, mean over study dates			
Study	Substudy	Mean ± SD	Mean ± SD	*r̄_j_*	SD	T (°C)	DP (°C)	SLP (kPa)	RH (%)
Wallace 1996		42.6 ± NR	70 ± NR	0.41	0.16	11.7	52.0	101.81	27.3
Rojas-Bracho et al. 2000		14.2 ± 11.2	21.6 ± 13.6	0.64	0.11	13.2	45.4	101.56	68.0
Williams et al. 2000a, 2000b		22.0 ± 12.0	13.0 ± 3.2	0.79	0.08	24.0	64.0	101.85	68.3
Rodes et al. 2001	1	20.5 ± 13.4	13.1 ± 5.9	0.58	0.18	9.6	41.8	102.27	75.2
	2	10.1 ± 3.2	11.1 ± 2.8	0.65	0.20	17.5	41.2	101.42	43.9
Suh et al. 2003	1	19.3 ± 9.0	25.1 ± 20.8	0.32	0.14	21.1	60.3	101.34	71.3
	2	13.5 ± 8.5	19.6 ± 14.5	0.59	0.16	13.7	46.8	101.70	69.7
Liu et al. 2003	1	9.0 ± 4.6	9.3 ± 8.4	0.47	0.10	9.9	43.6	101.78	78.9
	2	9.2 ± 5.1	10.5 ± 7.2	0.51	0.09	10.8	44.8	101.78	77.8
	3	12.6 ± 7.9	10.8 ± 8.4	0.55	0.13	10.0	42.8	101.82	76.0
	4	11.3 ± 6.4	13.3 ± 8.2	0.41	0.11	6.9	37.8	101.90	77.1
Reid 2003	1	14.5 ± 7.3	16.3 ± 8.4	0.76	0.18	15.7	49.7	102.01	68.3
	2	22.7 ± 10.6	15.0 ± 7.5	0.48	0.12	17.2	49.8	101.64	62.0
Williams et al. 2003a		19.3 ± 8.43	23.0 ± 16.1	0.35	0.04	17.2	51.9	101.92	67.4
Brown et al. 2008	1	8.6 ± 5.2	12.0 ± 6.0	0.25	0.22	2.0	22.7	101.67	59.0
	2	12.5 ± 7.6	10.0 ± 6.2	0.75	0.35	20.4	58.6	101.43	70.3
All nine studies totaled 1996–2008	16	13.9 ± 7.9	13.2 ± 8.2	0.53	0.14	13.4	46.1	101.78	69.0

Abbreviations: DP, dew point; NR, not reported; *r̄_j_*, mean within-participant residential outdoor PM_2.5_-personal PM_2.5_ correlation coefficient; RH, relative humidity; SD, standard deviation; SLP, sea level pressure; T, temperature.
